# Lignocellulosic Fibers from Renewable Resources Using Green Chemistry for a Circular Economy

**DOI:** 10.1002/gch2.202000065

**Published:** 2020-11-04

**Authors:** Khandoker S. Salem, Ved Naithani, Hasan Jameel, Lucian Lucia, Lokendra Pal

**Affiliations:** ^1^ Department of Forest Biomaterials NC State University Raleigh NC 27695–8005 USA

**Keywords:** bamboo, carbonate hydrolysis, green chemistry, hemp, high‐kappa kraft pulping, sustainable fibers

## Abstract

The sustainable development of lignocellulose fibers exhibits significant potential to supplant synthetic polymer feedstocks and offers a global platform for generating sustainable packaging, bioplastics, sanitary towels, wipes, and related products. The current research explores the dynamics of fiber production from wood, non‐wood, and agro‐residues using carbonate hydrolysis and a mild kraft process without bleaching agents. With respect to carbonate hydrolysis, high yield, and good coarseness fibers are attained using a simple, low‐cost, and ecofriendly process. Fibers produced using a mild kraft process have lower Klason lignin, carboxyl content, surface charges, and higher fiber length, and crystallinity. Eucalyptus fibers show the highest crystallinity while softwood carbonate fibers show the lowest crystallinity. Hemp hurd fibers contain the highest concentration of hard‐to‐remove water, and thus, suffer maximum flattening visualized by the microscopic images. The relatively high yield sustainable fibers with versatile properties can provide a significant economic benefit since fiber is the dominant cost for producing various bioproducts to meet society's current and future needs.

## Graphical abstract

Sustainable innovations in lignocellulosic fibers are paramount for high‐value bioproducts such as packaging, composites, tissue and hygiene, and molded products amongst others in a circular economy to address the global challenges of health, food security, and environment.

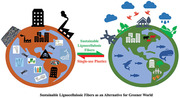

## 1 Introduction

The world has been moving towards a sustainable, bio‐based and circular economy due to social awareness and governmental regulations against single plastic use which could save up to $26 trillion by 2030.^[^ ^1^, ^2^ ^]^ Global environmental awareness has propelled the bio‐based industries, like paper, tissue paper products, bioplastics, packaging, renewable lubricants, etc., more so than many other industries to play an important role in sustainable development because among their chief raw materials are renewable wood and non‐wood fibers.^[^ ^3^, ^4^ ^]^ Appropriate and sustainable utilization of bioresources are the major requirements to expand the bio‐based economy to produce biochemicals, biofuels, bioplastics, and related products to their petroleum analogues. Wood has been a major source of biomass for the places which are rich in forestry resources. But, in places where the availability of forest is limited, non‐wood species and agro‐industrial waste can be possible alternatives and equally valuable sources of biomass. However, the foremost challenge in producing sustainable lignocellulosic fibers is ensuring a sustainable source of biomass supply, since lignocellulosic resources are prima facie feedstocks for pulp, lignin, nanocrystals nanofibers, and biopolymers, which have versatile industrial applications, for example, paper products, bioplastics, biocomposites, biofuels, and bioenergy production, etc. Indeed, lignin has emerged as a potential sustainable bioresource for advanced applications due to high carbon content and renewable character.^[^ ^5^ ^]^ Researchers have developed techniques to valorize lignin for applications in polymers and composites^[^ ^6^, ^7^ ^]^ and for producing sustainable thermo–electric materials based on carbon nanofibers (CNFs).^[^ ^8^, ^9^ ^]^ These CNFs have been successfully used as an alternative to carbon nanotubes (CNTs) to create high performance anodes for better thermo‐mechanical and electrical properties.^[^ ^10^, ^11^ ^]^ However, to continue to increase its utilization, the circular economy is pivotal though it has been very difficult to efficiently recycle lignocellulosic fibers due to limitations in collection and its high degree of contamination. Thus, extensive research is being carried out to produce sustainable feedstock by introducing alternative pulping methods and/or alternative raw materials that would conserve the usage of chemicals, energy, and water.^[^ ^12^, ^13^, ^14^ ^]^

Despite considerable research effort, wood is still the predominant resource for pulp production because it has been used to produce 89% of the world's paper, whereas, only 11% is based on non‐woody plants.^[^ ^15^ ^]^ However, non‐woody and agro‐residues such as hemp hurds have been emerging as a viable resources to meet growing demand for economic and sustainable paper products.^[^ ^16^ ^]^ Presently, hemp hurd has been used to meet 5% of the total global paper supply for tissue papers, bio‐plastics etc., which has been made possible due to the valorization of the hemp hurd from its original use as animal bedding and hemp‐lime construction materials.^[^ ^16^ ^]^ The high cellulose content ≈ 50%–70%, quick harvesting time (4 months) and requirement of fewer harsh chemicals for processing, with respect to the hardwoods and softwoods, have made hemp hurd a promising candidate for sustainable lignocellulosic resource.^[^ ^16^ ^]^

Another growing challenge for sustainable fiber sources is the introduction of an environment friendly, low chemical pulping methods to raise pulp yield. Despite considerable research effort, kraft and soda are still the predominant processes for producing fibers with low lignin content suitable for papermaking.^[^ ^17^ ^]^ Almost 80% of the total chemical pulping industry employees kraft pulping, which involves digestion of wood chips in a solution of sodium sulfide and sodium hydroxide at elevated temperatures and pressures.^[^ ^18^ ^]^ It offers the benefit of recovering the cooking chemicals and heat, but also has some drawbacks including emissions of sodium and calcium salts particulates (flue gases) and volatilized reduced sulfur compounds.^[^ ^19^, ^20^, ^21^, ^22^, ^23^, ^24^ ^]^ In contrast to kraft, soda and organosolv are sulfur‐free processes and thus alkali such as NaOH, KOH, and Ca(OH)_2_ are widely used as pretreatment steps for pulping.^[^ ^25^ ^]^ They can be performed at ambient conditions, but require longer pretreatment times.^[^ ^26^, ^27^ ^]^ Alkali pretreatment is most effective for biomass having low lignin contents such as leftover lignocellulosic feedstocks, corn stover, switchgrass, bagasse, wheat, and rice straw.^[^ ^28^ ^]^ Organosolv has been proposed based methanol, ethanol, acetic acid, acetone, etc., to remove lignin.^[^ ^29^ ^]^ Although this technique comes with reduced emissions of sulfur dioxide and odorous gases because of the use of sulfur‐free technology, it is not ready yet to be implemented on an industrial scale.^[^ ^30^ ^]^ Thus, there is a great opportunity for an alternative pulping system to reduce ecological damage by minimizing waste in conventional pulping.^[^ ^24^ ^]^

The current work discusses new types of chemical pulping where significant efforts have been made to attain sustainable utilization of natural resources. The new processes were carried out using very mild chemical conditions and resulted in higher yield of pulp compared to conventional methods (34%–55%).^[^ ^31^ ^]^ Non‐conventional pulping using 4% sodium carbonate based on Na_2_O for carbonate hydrolysis and 12% active alkali (NaOH + Na_2_S, based on Na_2_O) for mild kraft were studied for five feedstocks: eucalyptus, hemp hurd, bamboo, hardwood and softwood. Fiber yield, lignin content, brightness, surface charges, hard to remove water, and crystallinity were determined. Hence, the present work demonstrates how carbonate hydrolysis and mild kraft pulping can produce higher value fibers. This study will enable acceleration of the biorefinery to tune the properties different feedstock fibers.

## 2 Experimental Section

### 2.1 Materials

Five types of feed stocks were selected: Futura 75 cultivar hemp hurds, dew retted, and decorticated procured from the Netherlands and Bamboo (Henon) supplied by National Bamboo, LLC. Raleigh, NC, Southern pine, eucalyptus (*E. grandis*), and mixed hardwood and softwoods from local mills. The chemical compositions of these fibers were determined according to the guidance of U.S. Department of Energy, National Renewable Energy Laboratory (NREL) analytical procedure TP‐510‐42618 or the ASTM E1758‐01 (2015) in agreement with previous studies^[^ ^32^, ^33^ ^]^ presented in **Table** **1**. All chemical reagents were purchased from Fisher Scientific.

**Table 1:** Chemical composition of feedstock

Raw material	Cellulose [%]	Klason lignin [%]	Extractives [%]	Ash [%]	Hemicelluloses [%]
Eucalyptus	46.7	27.9	4.3	1.3	19.8
Hemp hurds	43.0	24.4	2.2	1.4	29.0
Bamboo	43.2	26.7	2.2	0.8	27.1
Hardwood	44.6	26.9	3.7	1.1	23.7
Softwood	46.0	28	3.0	1.0	22.0

** Constituent content calculated on a dry basis by subtracting sum of cellulose, lignin, extractives, and ash contents from 100%.

### 2.2 Methods

#### 2.2.1 Defibration

**Figure 1:**
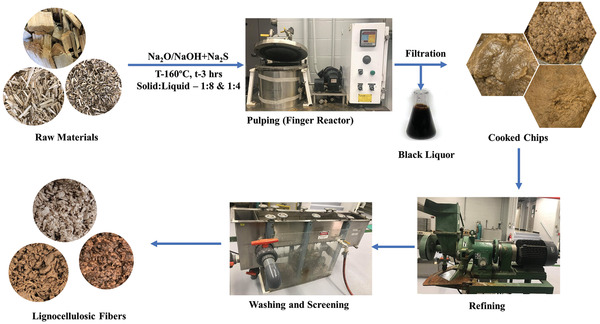
The pulping scheme for different feed stocks and subsequent processing of fibers.

Laboratory defibration highlighting the features of sodium carbonate and mild kraft defibration are shown in **Figure** **1**. The defibration process conditions are in **Table** **2**. A higher water‐to‐solids ratio of 8:1 was used for hemp due to its low bulk density compared to hardwood. The carbonate hydrolysis was carried out using 4% carbonate (based on Na_2_O). Kraft pulping was done using 12% active alkali and 25% sulfidity (NaOH+Na_2_S) (all on an Na_2_O basis). Both processes were conducted in a stainless‐steel reactor under controlled temperature (160 °C) for 3 h using a mild concentration of sodium carbonate (carbonate) and a high concentration of alkali charge (kraft), respectively. The pulp was washed and refined on the laboratory disc refiner (The Bauer Bros Co., Springfield, Ohio, Model 148‐2, rpm 3600) at disc gaps of (0.2–0.1–0.05) mm with three passes for hard wood pulp and disc gaps of (0.1–0.05) mm with two passes for hemp pulp before screening on a 0.15 mm slotted laboratory screen. Pulp yield, Klason lignin content, and freeness were determined using the TAPPI T222 and TAPPI T227 om‐09 standard methods, respectively.

**Table 2:** Process condition

Process	Feedstock	pH	Water–solid ratio	Pulping chemicals
Carbonate hydrolysis	Hemp	11.2	8	4% sodium carbonate based on Na_2_Oa)
	Eucalyptus, bamboo, hardwood, softwood	11.3	4	4% sodium carbonate based on Na_2_O
Mild kraft	Hemp	12.8	8	12% active alkali 25% sulfidity (NaOH+Na_2_S) based on Na_2_O
	Eucalyptus, bamboo, hardwood, softwood	13.3	4	12% active alkali 25% sulfidity (NaOH + Na_2_S) based on Na_2_O

^a)^ Reporting on a Na_2_O basis indicates the actual chemical relationship between the pulping chemicals used. It is a standardized way of normalizing the concentration strengths (based on Na_2_O).

For simplicity, the following sample IDs will be mentioned in the later part of the article: EU_C and EU_K (Eucalyptus fiber produced by Carbonate and Kraft process); HM_C and HM_K (Hemp fiber produced by Carbonate and Kraft process); BMB_C and BMB_K (Bamboo fiber produced by Carbonate and Kraft process); HW_C and HW_K (Hardwood fiber produced by Carbonate and Kraft process) and SW_C and SW_K (Softwood fiber produced by Carbonate and Kraft process)

#### 2.2.2 Characterization of Different Fibers

##### Fiber Quality Analysis

Fiber length (*l*_w_), fine contents and other physical properties of the different samples were determined by using a high‐resolution fiber quality analyzer: HiRes FQA, OpTest Equipment Inc, Hawkesbury, ON, Canada. Before testing, fiber quality analysis (FQA) was calibrated and used according to the manufacturers’ specifications. The whole pulp for each sample was taken and disintegrated before the measurement was done.

##### Fourier Transform Infrared Spectroscopy (FTIR)

Fourier transform infrared (FTIR) spectra of the different fibers were recorded using a PerkinElmer Spectrum One FTIR spectrometer. For each sample, the diamond crystal of an attenuated total reflectance (ATR) accessory was brought into contact with the area to be analyzed. The contact area was a circle of about 1.5 mm in diameter. All spectra were recorded between 4000 and 500 cm^−1^ with a resolution of 4 cm^−1^ with 32 scans per sample. For comparison, spectra were adjusted to the same baseline.

##### Brightness Measurement

The brightness of the fibers was measured by following the ISO 2470 standard method using the color spectrophotometer Color Touch X, Technidyne, Indiana, USA. This procedure was repeated for ten samples for each of the fibers, and the mean value was taken.

##### Total Carboxyl Content Analysis

Acid‐base titration was used to determine the total carboxyl content of the pulps.^[^ ^34^, ^35^ ^]^ 100 mg of pulp was dissolved in 20 mL 0.1 n NaOH and stirred with a magnetic stirrer for 2 h. An excess amount of 0.1 n NaOH was titrated with 0.1 n HCl using phenolphthalein as an indicator. The carboxyl content in milliequivalents per 100 g of the pulp slurry was calculated as:

(1) $$\begin{array}{c} \text{Carboxyl}\;\text{content}=\left(V_{b}-V_{a}\right)\times N\times 100/W \end{array}$$

Where, *N* is the normality of HCl (Equation (l)), *V*_b_ and *V*_a_ are the volumes of HCl in absence and presence of sample, and *W* is the weight of sample (g).

##### Charge Determination

Charge estimation on the fibers was carried out using the colloidal titration method.^[^ ^36^ ^]^ The fiber samples were disintegrated into pulp at a 0.075% consistency (mass of solids relative to total mass + water) in deionized water. The colloidal charge of 200 mL of the resulting pulp suspension was evaluated by titration using a CHEMTRAC ECA 2000 P streaming current analyzer. The charge neutralization points of fibers were determined by the addition of cationic polymers (poly(diallyldimethylammonium chloride) (polyDADMAC)) to the suspension.

##### Hard‐to‐Remove Water Measurement

Hard to remove (HR) water was determined using TGA.^[^ ^37^ ^]^ The sample was taken in platinum sample pans. The temperature was then raised to 110 °C from 30 °C at 10 °C min^−1^. Drying experiments were run isothermally at 110 °C for 15 min until no more drying occurred. The non‐freezing bound water was calculated by subtracting the total freezable water from the moisture ratio in the sample determined gravimetrically using a TGA microbalance (110 °C until weight did not change).

##### X‐Ray Diffraction (XRD) Analysis

A Rigaku SmartLab X‐Ray Diffractometer, operated at 45 kV and 40 mA with a Ni‐filtered CuK radiation, was used to determine the crystallinity index of the samples. X‐ray diffractograms were recorded at 0.02° s‐1 over a 2 scan in the range 5°–60°. Segal Peak height method was used to calculate the crystallinity index (CI) of the samples using the following equation:

(2) $$\begin{array}{c} \text{Crystallinity}\;\text{Index}\;\left(\text{CI}\right)=\left(I_{002}-I_{\text{AM}}\right)\times 100/I_{002} \end{array}$$

##### Scanning Electron Microscopy (SEM)

Handsheets of the different fibers produced using carbonate and mild kraft pulping process were made at a target basis weight of ≈40 g m^−2^ as per the TAPPI T205 method with a light weight (≈0.15 kg) foam roller instead of a standard heavy (13 kg) brass roller, without any pressing, and dried twice on a drum dryer at 220°F. The morphological characterization of samples was carried out using Hitachi S3200N variable pressure scanning electron microscope (VPSEM). Samples were sputter coated with AuPd coating for 10 minutes and images were taken at 10kV.

## 3 Results and Discussions

### 3.1 Fourier Transform Infra‐Red Spectroscopic Analysis

**Figure 2:**
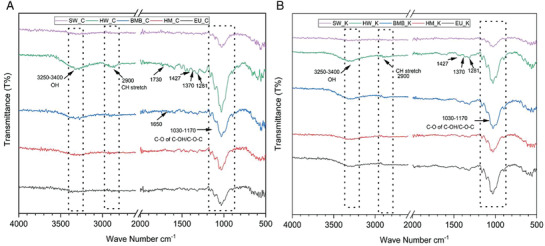
FTIR spectra of different fibers prepared by A) Carbonate Hydrolysis and B) Mild Kraft.

The chemical structure of the different fibers prepared by carbonate hydrolysis and kraft process were compared in **Figure** **2** to examine the effect of different pulping on the fiber structures. The appearance of peak in the region of 1030–1170 cm^−1^ is due to C—O—C and C—O stretch of primary and secondary hydroxide groups of the carbohydrates and hydroxyphenyl, guaiacyl, and syringyl groups of lignin.^[^ ^38^ ^]^ The peaks at 1281, 1370, and 1427 cm^−1^ may be due to aromatic esters, ether, and phenol compounds.^[^ ^39^ ^]^ The peak corresponding to 1730 cm^−1^ is from unconjugated C—O stretching which is due to vibration of aliphatic carboxylic acids and ketones of hemicellulose or lignin groups and that near 1650 cm^−1^ is due to conjugated carbonyl found in hemicellulose and lignin groups.^[^ ^40^ ^]^ Moreover, CH symmetric and asymmetric stretching bands appeared at 2900 cm^−1^ and hydrogen bonded stretching bands of OH groups at 3400 cm^−1^ are characteristics peaks of hydroxyl groups of cellulose.^[^ ^39^ ^]^ All the fibers, including the hemp hurds, had similar FTIR spectra since they had similar lignocellulosic compositions as shown in Table 1 and none of the pulping resulted in significant change in the chemical structure of the fibers.

### 3.2 Pulp Yield

**Figure 3:**
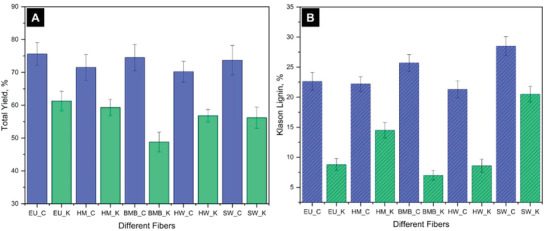
Graphical representation of A) Total yield and B) Klason lignin content of different fibers produced using two different methods.

The yield of the carbonate and kraft pulps (total pulp wt% obtained after defibration) were calculated and found to vary from 49%–75% depending on the source of fibers and pulping, which were comparatively higher than the pulp yield produced by conventional methods as shown in **Figure** **3**A. The yield of carbonate hemp pulps was 71.3%, which was slightly less than the carbonate eucalyptus and bamboo, but almost the same as carbonate hardwood and softwood pulps. However, the yield of mild kraft hemp was similar to eucalyptus but higher than the other kraft pulps. This might be due to the morphology of the hemp hurds allowing easier penetration of pulping chemicals compared to hardwood and softwood.^[^ ^16^ ^]^ The pulp yield was higher for the carbonate than the kraft for all the fiber types because carbonate was operated at lower pH. Higher pH resulted in higher hydroxyl concentration which controls the intensity of fiber defibration and consequently causes random chain scission in cellulose and hemicelluloses. This promotes peeling and results in lower pulp yield because the pulp defibration rate is proportional to the —OH concentration.^[^ ^16^, ^41^, ^42^ ^]^ Thus, kraft pulping facilitated removal of higher amounts of lignin at high pH as shown in Figure 3B and supported by FTIR (Figure 2B). The peaks corresponding to 1730 and 1650 cm^−1^ arise from lignin functional groups from carbonate which is less visible in mild kraft because lignin content is relatively low in the latter. Thus, carbonate hydrolysis and mild kraft produced pulps with different yields, lignin percentages, and different compositions.

### 3.3 Fiber Morphology

The fiber structure was further investigated by comparing the fiber morphology of the pulps produced by the two processes. Fiber morphology is related to fiber structure, which is primarily regulated by feedstock and pulping. Generally, fiber length, coarseness, kink, curl and fines are used to describe fiber morphology. **Table** **3** provides data on the morphology of fibers.

**Table 3:** Physical Properties of Different fibers

Sample ID	Fiber length *L*_w_, [mm]	Coarseness [mg m^−1^]	Curl index	Kink index [1 mm^−1^]	Fines [%]	Brightness ISO
EU_C	1.06	0.20	0.05	0.67	2.29	10.9
EU_K	1.09	0.08	0.05	0.08	2.12	22.5
HM_C	0.69	0.18	0.07	1.27	7.30	15.7
HM_K	0.77	0.07	0.05	0.08	3.93	23.4
BMB_C	1.22	0.36	0.07	0.80	17.73	10.9
BMB_K	1.41	0.11	0.06	0.62	15.09	23.3
HW_C	0.8	0.20	0.08	0.84	5.81	12.7
HW_K	1.17	0.10	0.05	0.62	2.33	19.7
SW_C	1.94	0.45	0.06	0.65	4.29	12.6
SW_K	2.45	0.27	0.05	0.55	1.25	13.2

The length of all the fibers produced by kraft pulping increased; but the other properties: coarseness, kink, curl, and fines showed an opposite trend, that is, decreased when compared with the fibers produced by carbonate. The production of longer fiber and lower fines during kraft can be attributed to higher pH. Higher pH resulted in longer fibers, lower coarseness, and lower fines by removing more hemicelluloses, lignin, and extractives as reported earlier.^[^ ^16^, ^43^ ^]^

Studies of fiber properties have shown that the fiber strength increases with increases in fiber length and decrease in coarseness. Longer fibers have more fiber joints and a higher area for bonding and therefore create a stronger network compared to shorter fibers. On the other hand, thinner fibers collapse more easily than a coarser fiber with thicker cell walls. The collapsed fibers will create a network with much higher density and lower bulk. The flexibility of the fibers and the number of fiber joints will therefore increase the tensile strength. The uncollapsed fibers will create a network with higher porosity and higher bulk compared with the collapsed ones.^[^ ^44^ ^]^ Thus, fibers produced in kraft will form stronger sheets and possess better tensile properties.^[^ ^45^ ^]^ However, fiber curliness negatively affects tensile and tensile stiffness indices, while it increases tear and fracture toughness indices.^[^ ^46^ ^]^

The curl index gives the maximum elongation of the fiber up to breakage, which indicates the stability of the paper web in the open draw. The deformed fibers contribute uneven distribution of stress along the length of a curled fiber in a fracture zone, transferring more stresses to the bonds, which increases the tear strength of the sheets. Thus, fibers produced by carbonate form sheets having lower tensile strength, but higher tear strength. The fiber curl, in addition, increases the bulk and porosity of the pulp sheet but decreases the drainage resistance.^[^ ^46^ ^]^ Curly fibers cause more scattered reflection of light, which results in a matte appearance and opacity. Thus, fibers produced by carbonate have higher coarseness and lower brightness than kraft. The presence of lower lignin content in kraft also affected the brightness. Thus, carbonate pulps with higher coarseness, curl index and fines could be used to make paper or tissue products with high bulk and tear strength, whereas, kraft pulp generates longer, brighter fibers to give stronger, flexible and brighter paper products.

### 3.4 Carboxyl Content and Surface Charge

**Figure 4:**
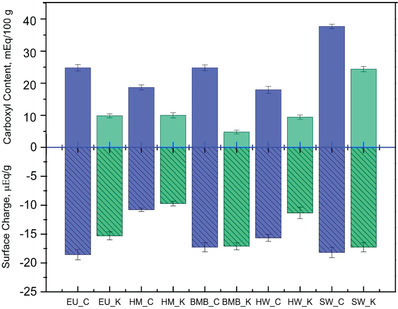
Carboxyl content and surface charge of different fibers produced using two different methods.

In addition to the above mentioned properties, the charge on fibers is a well‐known parameter in papermaking and plays a vital role in their performance through electronic interactions of the charged soluble and particulate fractions.^[^ ^43^ ^]^ The electrical charges at the surfaces of the cellulose fibers are a significant parameter strongly affecting the swelling ability of cellulose fibers and provides a driving force for adsorption of retention aids, sizing agents, and strength enhancing chemicals.^[^ ^47^ ^]^ Fiber charge is a significant characteristic of cellulose fibers, which strongly affects post‐processing such as enzymatic modification.^[^ ^48^ ^]^ The carboxyl content and surface charge of different fibers are shown in **Figure** **4**. The carbonate pulps have relatively higher carboxyl content and surface charge than kraft. The carboxyl content of the fibers is formed due to conversion of the 4‐O‐methylglucuronic acid side groups (MeGlcA) of xylan to hexenuronic acids (HexA) by elimination of the 4‐Omethoxyl group and deprotonation during peeling at alkaline conditions.^[^ ^47^ ^]^

The carboxyl content of hemp pulp produced by carbonate was similar compared to carbonate hardwood pulp, but lower than the other carbonate pulps. However, the kraft hemp pulp contained almost similar carboxyl content like eucalyptus and hardwood but lower than the bamboo and softwood pulps produced by mild kraft. The lower carboxyl content of the kraft pulp is due to higher alkaline conditions at which the pulping was carried out which decreased reprecipitation of lignin and hemicellulose onto the surface of the fibers.^[^ ^47^ ^]^ The relatively higher surface charge of the carbonate pulps was due to its high anionic charge density contributed by resins, fatty acids, carboxylic acid groups of the hemicelluloses, and lignin's phenolic groups.^[^ ^49^ ^]^ The hydrophilic carboxyl group in the fiber interacts with water and enables fibers to absorb more water to increase swelling capacity, important for the tissue paper industry.^[^ ^50^ ^]^ The presence of greater numbers of carboxyl groups and surface charge in the fiber facilitates better entanglement by inducing hydrogen bonding to increase the tensile and burst properties of the fibers.^[^ ^44^, ^51^ ^]^ The carboxyl groups act as binding sites and also enables controlled chemical loading and release by regulating the interactions between the carboxyl group and chemicals.^[^ ^34^, ^35^ ^]^ Thus, tissue, towels and wipes with varying surface charge and carboxyl content can be produced by using different fiber types or pulping (Figure 4).

### 3.5 Crystallinity Indices

**Figure 5:**
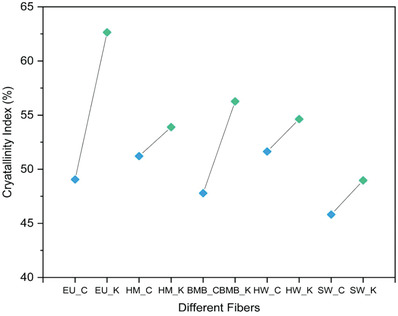
The Crystallinity Index of different fibers produced using two different methods.

The effect of the carbonate and mild kraft pulping on the crystalline structure was evaluated further to study the changes in crystallinity index of different fibers. It was found that the crystallinity indices of the kraft fibers were higher than that of carbonate fibers as shown in **Figure** **5**. The amorphous nature of the fiber is imparted by the hemicelluloses which is a short, linear and highly branched chain of sugars and possesses shorter side chains.^[^ ^52^ ^]^ Thus, they induce a branched network through extensive crosslinking to lignin and cellulose by cinnamate acid ester linkages and hydrogen, respectively.^[^ ^53^ ^]^ The kraft fibers has higher crystallinity since the process was operated at higher pH which facilitated dissolution of a higher level of amorphous cellulose. pH range at which carbonate ruptured the structural linkages between lignin and other carbohydrate fractions in the lignocellulosic biomass, but could not remove lignin as much as kraft.^[^ ^54^, ^55^ ^]^ As a result, the carbonate treatment of the fibers resulted in a carbohydrate heterogeneous matrix with lower crystallinity compared to kraft and a lower degree of polymerization.^[^ ^47^ ^]^

### 3.6 Hard‐to‐Remove Water

**Figure 6:**
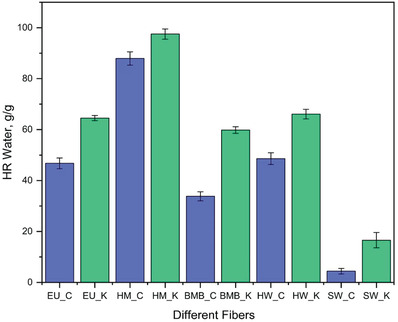
Hard to remove water content of different fibers.

The Hard‐to‐Remove water is defined as trapped water, freezing bound water, and non‐freezing bound water which requires a higher energy for drying.^[^ ^37^ ^]^ It is an important parameter as it affects the different properties of the paper products like water absorption, tensile, drying energy, and the reactivity cellulose towards chemicals.^[^ ^37^, ^44^, ^56^ ^]^ Thus, the effect of the different pulping processes on the HR water content of the different feedstocks were investigated graphically represented in **Figure** **6**.

**Figure 7:**
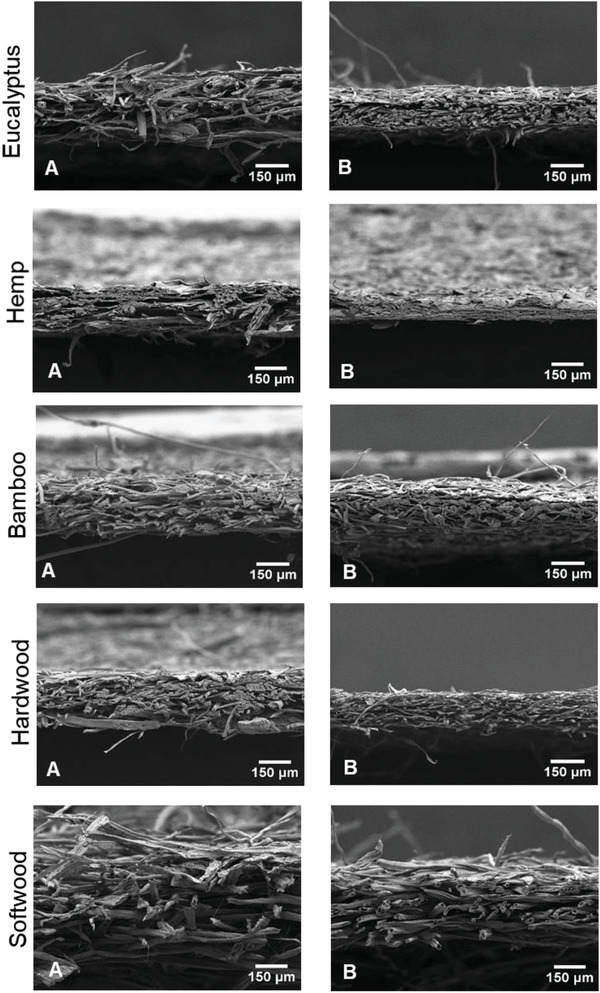
SEM images of different fibers produced using A) Carbonate Hydrolysis and B) Mild Kraft techniques.

It can be observed form Figure 6 that the hemp fiber produced using carbonate and mild kraft contained the highest levels of HR water compared to other fibers, whereas the carbonate and kraft softwood fibers contained the least amount of HR water. The hemp fiber had higher HR water than the others since it has a much higher hemicellulose content. Hemicellulose is amorphous in nature and allows more water penetration which facilitates more fiber‐water interaction increasing the HR water content.^[^ ^52^, ^56^ ^]^ The presence of relatively higher lignin content in softwood reduces the HR water content as lignin being hydrophobic diminishes fiber‐water interactions.^[^ ^57^ ^]^ However, HR water content was higher for fibers produced using kraft (Figure 6). The carbonate fibers, though they have less crystallinity, are associated with less HR water. This might be due to the presence of higher lignin content which limits the ability of water molecules to form hydrogen bonds with the hydroxyls of the cellulose.^[^ ^58^ ^]^ On the contrary, kraft fibers have relatively much less lignin content which allows the cellulosic hydroxyls to interact with the water forming hydrogen bonds and increasing HR water content. Thus, kraft fibers would collapse more as shown in **Figure** **7** because the HR water regulates extent of fiber collapsing. The reactivity of the kraft fibers will be negatively affected as well, since HR water hinders the facile diffusion of chemical reagents by forming a hydration barrier which limits accessibility to the hydroxyl groups and reduces the reaction kinetics.^[^ ^56^ ^]^ Thus, lower HR content of the carbonate fibers indicates that they will suffer less hornification than kraft fibers, supported by the coarseness data of the fibers as shown in Table 3 and SEM images in Figure 7.

### 3.7 Scanning Electron Microscopic Image Analysis

SEM image analysis was done to visualize the effect of carbonate and mild kraft on fiber morphology. It can be observed form the images of the different fibers that the handsheets made from kraft suffer greater flattening than carbonate (Figure 7). These results directly relate to the coarseness and HR water of the fibers. Higher bulk of carbonate handsheets can be attributed to higher coarseness of the pulps produced by carbonate. Thus, kraft hemp pulp having the least coarseness (Table 3) yielded sheets with the least thickness (Figure 7B), whereas, the carbonate softwood showed highest thickness owing to its highest coarseness.

The kraft fibers exhibited higher HR water and as HR water leaves during drying, more hydroxyls of cellulose become available for bonding. These hydroxyls interact with each other forming hydrogen bonds which leads to irreversible flattening of the fibers resulting in higher flattening of the fibers.^[^ ^56^ ^]^ Therefore, hemp fibers suffered the highest flattening due to its least coarseness (Table 3) and highest HR water value (Figure 6).

## 4 Conclusions

This study reports production of pulp from several fiber feedstocks after sodium carbonate and mild kraft pulping processes. The pulp yield was higher for sodium carbonate, which is a significant economic benefit because fiber is the dominant cost for producing paper products. The high yield of hemp fibers using both pulping processes and its low coarseness, low fines, and high brightness value qualify it an emerging potential resource for paper and tissue paper industries because it improves tensile, burst resistance, and softness of tissue handsheets. The lignin content was lower for pulp produced using mild kraft because it removes more lignin, which results in decreased coarseness. Higher coarseness and lower HR water content using carbonate led to less fiber collapsing, very useful for maintaining higher bulk for tissue paper products which has a direct correlation with water absorbency and bulk softness. Moreover, a lower HR water content of carbonate also facilitates chemical treatment of the pulp at much less chemical consumption compared to kraft because the HR water acts as a barrier for chemical modification. The kraft pulps, on the other hand, have longer fibers, lower curl and kink index, and higher brightness, important for producing strong sheets. The carbonate pulps possessed higher carboxyl and surface charge which increases water uptake, a very important requirement for paper towels. Thus, carboxyl content can be controlled using different pulping methods and fiber types, which can be manipulated to control the binding capacity of the fibers and have potential use in antimicrobial tissue wipes.

## Conflict of Interest

The authors declare no conflict of interest.
